# Types, norms, and normalisation: Hormone research and treatments in Italy, Argentina, and Brazil, c. 1900–50

**DOI:** 10.1177/0952695120941193

**Published:** 2020-09-17

**Authors:** Chiara Beccalossi

**Affiliations:** 4547 University of Lincoln, UK

**Keywords:** Argentina, biotypology, Brazil, hormones, Italy

## Abstract

Displacing the physiological model that had held sway in 19th-century medical thinking, early 20th-century hormone research promoted an understanding of the body and sexual desires in which variations in sex characteristics and non-reproductive sexual behaviours such as homosexuality were attributed to anomalies in the internal secretions produced by the testes or the ovaries. Biotypology, a new brand of medical science conceived and led by the Italian endocrinologist Nicola Pende, employed hormone research to study human types and hormone treatments to normalise individuals who did not conform to accepted medical norms. Latin American medical doctors, eugenicists, and sexologists took up biotypology with enthusiasm. This article considers the case studies of Italy, Argentina, and Brazil, and analyses the work of medical doctors who adopted a biotypological mode of reasoning and employed to various extents hormone therapies in their practice. By focusing on hormone therapies that aimed to normalise secondary sexual characteristics and the sexual instinct, the article suggests that while the existence of normality was contested to the point that a number of medical scientists argued that no such thing existed, the pursuit of normality was carried out in very practical terms through the new medical technologies hormone research had introduced.

In *Discipline and Punish*, Michel Foucault took the concept of normalisation to refer to an idealised norm of conduct, which was increasingly imposed through institutions such as prisons, schools, and asylums (Foucault, 1991[1975]). In other works, Foucault suggested that normalisation techniques proliferated in the 19th century, as the state expanded, and statistical sciences, eugenics, and sexology came into being (Foucault, 1998[1976]). In his account, the ‘optimisation’ of the body’s ‘capabilities’ is central to modern medicine, an analysis developed more recently by Nikolas Rose (2007). Foucault directed scholars’ attention to the strategies of biopolitics: the expansion of the scientific management of the population, along with the growth of eugenics, sexology, occupational health, and regimes of hygiene and beauty, all of this designed to create a more vigorous and healthy society so as to maximise efficiency, productivity, and governmental control over society (Foucault, 1998[1976]: 139).

Just a few generations before Foucault brought certain techniques and approaches to the attention of historians and other scholars, medical scientists were consciously and unabashedly pursuing the normalisation of the population. They developed new medical technologies to optimise and standardise the body’s functions in the interests of their own country and spoke openly about their mission to normalise people. In Southern Europe and Latin America, those medical scientists who first embraced constitutional medicine, and then biotypology – a medical classificatory science based on endocrinological research – devised new hormone treatments to normalise anomalous bodies and abnormal sexual behaviours. In doing so, they stressed that such normalisation would be of critical importance if governments wished to improve the ‘Latin race’.

Yet, as the history of normalisation through hormone therapies illustrates, the concept of the normal remained elusive. As this article will show, constitutional and biotypological studies in Southern Europe and Latin America seemed to suggest that there were endless human variations and that the normal body was merely an abstract concept, or an extremely rare occurrence in nature. This ambiguity stemmed in part from the adoption of 19th-century statistical methods that identified the normal with an ideal average. In their recent genealogical study on *Normality*, Peter Cryle and Elizabeth Stephens have rightly identified the central role of statistics in shaping the modern concept of normal ‘as an average that was also ideal, a typicality so perfect that no individual could ever embody it’ (Cryle and Stephens, 2017: 9). They argue that, despite the elusiveness and incoherence of the statistical concept of the normal, it was highly useful in other disciplines, from anatomy and medicine to the human sciences. The ‘conceptual incoherence’ of the idea of ‘normal’ was ‘the source of [its] enormous cultural productivity’ (ibid.).

By focusing on hormone therapies that aimed to normalise secondary sexual characteristics and the sexual instinct, this article suggests that while the existence of normality was contested to the point that a number of medical scientists argued that no such thing existed, the pursuit of normality was carried out in very practical terms through the new medical technologies hormone research had introduced. This article considers the case studies of Italy, Argentina, and Brazil and analyses the work of medical doctors who adopted a biotypological mode of reasoning and employed to varying extents hormone therapies in their practice. As I will explain below, biotypology was created in Italy and became particularly popular in Latin America. I have chosen the specific case studies of Italy, Argentina, and Brazil because my current research, which is still broader in scope, shows that there was a mutual exchange of sexual and endocrinological knowledge between these three countries in the interwar period. While medical knowledge travelled in both directions, from Italy to Argentina and Brazil and back to Italy, I have found less evidence that other countries where biotypology was well received, notably Mexico (Stepan, 1991), exported their sexual knowledge back to Italy.^1^ While exploring some aspects of the practical effects of hormone therapies, this article will pursue an intellectual history. Indeed, the aim of this article is to trace how types and norms were conceptualised by doctors who employed constitutional medicine and biotypology, and who studied how individuals who did not match gender and sexual norms were normalised. In this article, I am interested in the medical discourses behind the techniques of normalisation that shaped sexual knowledge in the interwar period in Italy, Argentina, and Brazil.^2^ For the sake of brevity, this article focuses on the work of selected individuals who are considered representative in their own country and who wrote about and used hormone treatments to normalise gender variants and individuals who were attracted to members of their own sex.

## Italy: Constitutional medicine and the emergence of biotypology

Ancient physicians had already admitted the existence of a correlation between body shape, the development of internal organs and their mode of functioning, personality, psychological attitudes, and a predisposition to certain disorders. First Hippocrates and then Galen identified four basic human groups, determined by the prevalence of one of the four humours: black bile, yellow bile, phlegm, and blood. Depending on which humour was dominant, an individual was believed to have a different temperament: melancholic, choleric, phlegmatic, or sanguine. In the Western world, medical thinking continued for centuries to embrace the idea of constitutional types; for example, it was still present within the neo-Hippocratism and neo-Galenism of the 17th century (Pende, 1939b: 16–46; Rossi, 1944: Vol. 1, 20–33). With the development of pathological anatomy in the 18th century, the humoral theory waned, but throughout the 19th century, modern medical schools that linked variations in the somatic proportions to a few basic human types reappeared – the so-called modern schools of constitutionalism.

In part, the development of modern constitutionalism was a reaction to laboratory medicine. Against the one-sided view of Pasteur’s medicine, which understood germs to be the sole cause of illnesses, and the localised view of diseases, medical men from different countries simultaneously raised objections: Achille De Giovanni in Italy, Friedrich Wilhelm Beneke in Germany, and Claude Sigaud in France, to name just a few, all favoured the cure of the individual as a whole organism (Rossi, 1944: Vol. 1, 31). Doctors who adhered to constitutional medicine believed that germs and lesions were necessary causes of sicknesses, but they were not sufficient to explain why some individuals got sick and others did not. As the evidence showed, not all individuals who were exposed to a germ, for example that of tuberculosis, developed the disease. Infection took root only in those people who were predisposed because of their constitution (Olby, 1994; Timmermann, 2001; Tracy, 1998). According to the advocates of constitutional medicine, the specific constitution, interpreted as the sum of the anatomical and functional characteristics of an individual, played a critical role in pathogenesis.

Modern constitutional medicine adopted a multifaceted approach to the clinic and varied from country to country. The so-called Italian School of constitutional medicine was particularly esteemed in both Europe and Latin America. It produced three generations of teachers and pupils who further advanced the discipline and defined and redefined the idea of type, normal type, and normality, using first anthropometry and statistics, and then hormonal formulae. More importantly for the argument developed here, this school produced a new science, biotypology, which employed the latest medical technologies, such as hormone treatments, to normalise individuals. The father of the modern Italian School of constitutional medicine was De Giovanni, professor of medical clinics at the University of Padua. In 1878, using anthropometric data that he had accumulated in his studies, he divided human beings into three different morphological types: (a) those with long limbs, a thin torso, and small diameters; (b) those with a developed torso, large diameters, and short legs and limbs; and (c) those with well-proportioned and harmonious measurements. Basically, De Giovanni described three constitutional human types according to the structure of the torso: brevilineal (short and fat), normotype (normal), and longilineal (tall and slim). In his *Morfologia del corpo umano* (Morphology of the Human Body; 1891), which represented the foundational text of modern constitutional medicine in Italy, De Giovanni explained that normality was an exception in real life. In the third part of this text, entitled ‘Method That Aims to Determine the Value of the Morphological Individual Type’, De Giovanni stated, ‘The morphological type, as commonly understood, the type that should represent normality in every respect and that should be found in the majority of people, does not exist. *The human morphological type, like the morphological type of race, is simply a concept*’ (De Giovanni, 1891: 131).^3^ He then clarified that, on the basis of his own studies, it was possible to identify the morphological characteristics that should be found in a ‘perfect type’, and it was therefore possible to delineate an ‘ideal type’. But in life, this type, or a type that was close to this ideal, was very rare. Indeed, in De Giovanni’s judgement, it was the exception that confirmed the rule (ibid.).

De Giovanni’s pupil, Giacinto Viola, refined the anthropometric method of his *maestro*, introducing statistics. Viola adopted the statistical method that the Belgian mathematician, astronomer, and statistician Adolphe Quetelet had developed in the 1840s, and used it to study the body’s morphology. Quetelet had suggested that human traits fell along a Gaussian curve, or normal distribution. The ‘normal’ was the average (Cryle and Stephens, 2017: 100–28). Viola, while adopting the basic type division of De Giovanni, believed that the normal type existed. By applying Quetelet’s statistical method to the study of individual constitutions, Viola came to the conclusion that the normal type, or ‘normotype’, was rare, admittedly, but did indeed exist in real life and that it manifested in 1.35% of the entire population. This ‘normotype’ was understood as the normal type in a statistical sense. According to Viola, the brevilineal and the longilineal constitutions were two opposite variations of the normal type (Pende, 1939b: 18). The latter had a harmonious body mass and proportions, but the normotype was not understood as a universal type. In Viola’s view, the body measurements of the normotype were the statistical average of the measures of the bodies of a specific region, and these normal measures, or this norm, changed in each region, and across different ethnic groups. Thus, within a country there were different norms, with factors such as nutrition and climatic conditions leading to alterations in this statistical norm (ibid.: 21).

In the interwar period, Italian constitutional medicine incorporated endocrinological research, and while employing hormonal formulae to study the body, it multiplied the constitutional types. Since the late 19th century, medical men who had worked on so-called ‘internal secretions’ of the body promoted the idea that an ideal healthy body was, in a fundamental sense, harmoniously regulated by the endocrine glandular system (Hausman, 1995: 27). Interestingly, endocrinology concerns itself with disorders within the human organism that are not caused by the invasion of germs and highlights the importance of the make-up of the entire body; not surprisingly, advocates of constitutional medicine displayed a strong interest in endocrinology, as both fields of study accorded great importance to the constitution.

The best example of how constitutional medicine changed as a result of the incorporation of endocrinology is the work of Nicola Pende, a well-known Italian fascist scientist. Graduating in 1903 with a thesis that demonstrated the relationship between the endocrine glands and the nervous system, Pende had been Viola’s medical assistant first in Palermo and then in Bologna. At the beginning of the 20th century, hormones were regarded as regulators of both tissue metabolism and corporeal forms, and Pende started to use his observations of how hormones functioned to divide human beings into types. Pende believed that hormone dynamics influenced metabolism in such a way that individuals could be classified as weak, or ‘asthenic’, and strong, or ‘sthenic’. Asthenic types had reduced muscle strength and functional deficiencies, and quickly reached exhaustion, while sthenic types exhibited good muscular strength and their organs functioned well. Developing Viola’s classification of human types but incorporating the asthenic and sthenic component, which was based on the way in which hormones functioned, Pende identified four basic deviations from the normal type: (a) the sthenic longilineal; (b) the asthenic longilineal; (c) the sthenic brevilineal; and (d) the asthenic brevilineal (Pende, 1933: 88). According to Pende, the normal or harmonious type (‘tipo equilibrato’) was also the average type (‘tipo medio’), and each type possessed a specific endocrine formula (ibid.: 61). Broadly speaking, Pende argued that the thyroid, the pituitary gland, and ‘certain genital and adrenal hormones’ stimulated the morphological differentiation of the body, while the parathyroids and the thymus and pineal glands stimulated more specifically the morphogenesis of the autonomic nervous system. When these two groups of hormones were ‘evenly balanced’, they produced the normotype. When the harmonic endocrine glands stimulating the autonomic nervous system were strong, the result was the brevilineal type. Finally, when the endocrine glands stimulating the morphological differentiation of the entire body predominated, the result was the longilineal (or ‘microsplanchnic’) type (Pende, 1928a: 66–7). This classificatory system, founded upon endocrine formulae, constituted the basis of Pende’s biotypology, his new version of constitutional medicine grounded in hormone research.

Pende himself coined the term *biotypology* in 1922 and, in his own words, biotypology was the science ‘of the architecture and engineering of the individual human body’ (Pende, 1939b: 1). The guiding assumption of this new science was that basic human types, or biotypes, existed, and that they all had different hormonal formulae. Each biotype had a body shape, as well as certain specific psychological characteristics and attitudes, and was likewise prone to certain diseases. Endocrine disorders, which were caused by an exacerbation, deficiency, or instability in the functioning of one or more endocrine glands, led to morphological, physical, and psychological disorders and so to a change from normal to abnormal conditions. Adhering to a venerable principle of natural philosophy stating that *natura non facit saltus*, Pende believed that normality and pathology were only quantitatively different and that their difference was merely one of degree (Pende, 1933: 33). In *Debololezze di costituzione* (Constitutional Inadequacies), by clarifying that ‘normal individuals’ and ‘healthy individuals’ were two separate questions, he defined normality as the statistical average:

Normality, in a statistical sense, is the mass of the average or central values—morphological, functional and psychological—of the curve of variability. But it is evident that, for we who are clinicians and biologists, the average *human type*—an abstract and ideal type that does not exist in Nature—is not the only type of healthy man. Many healthy subjects, we may even say the universality of healthy individuals, stray *more or less* from the statistical *average normal* type.…From the point of view of health and of functional robustness, wide deviations from the average type may exist. That is to say, individuals straying considerably from the normal average type—although within certain limits that cannot be established for all cases by bodily measurements—may nevertheless be healthy and functionally resistant. Accordingly, clinical normality, or health, and statistical normality, do not necessarily coincide. (Pende, 1928a: 50–2) (original emphasis)

Although the ideal type did not exist in nature, as Pende’s clinical experience showed, many subjects were, he continued, ‘nearest to the average type’ or ‘the type of Greek beauty’, which Pende believed embodied harmonious proportions (Pende, 1928a: 52).

Pende’s scientific output was massive, and he modified his views over time in the light of new endocrine discoveries. As a consequence, his approach to the classification of human beings, their bodies, and their temperaments changed as well. His taxonomy of human variations became more complex and the types multiplied over time. Broadly speaking, however, in different works Pende clarified that two criteria were critical for the definition of normality with reference to the human body: there was a ‘morphological statistical normality’, which varied across different regions of a country and across different countries, and there was an ‘aesthetic’ normality, which had its model in ancient Greece (Pende, 1939b: 381). Pende paid particular attention to regional and national variations of body types. In opposition to the kind of eugenics that had emerged in northern Europe – and in particular in Germany – Pende believed that there was no pure race, a fact that made it difficult to identify the average type. To him, a country such as Italy had been subject to so many waves of migration, invasions, and influences from other parts of the world over the centuries that the notion of a pure race seemed unrealistic.^4^ Yet each region in Italy had a population that could be considered homogeneous, and it was at a regional level that the average type could be found and needed to be studied and measured.

Different standards also defined the normal ideal female and male types. Addressing the issue of sexual differentiation and the sexual secondary characteristics, Pende assured other doctors that it was possible to assess the biometric indicators of femininity and masculinity. For women, such biometric indicators of ‘sound sexuality’ (‘buona sessualità’) included: the length of the thigh in relation to the length of the entire leg, the development of breasts, the width of the pelvis, the distribution of pubic hair, the voice, the menstrual cycle, and, of course, fecundity (Pende, 1939a: 27). In men, such indicators included genital volume, facial hair, a ‘masculine voice’, and musculature, in particular the strength of the neck muscles (ibid.: 27–8). He also revealed the specific normal biometric indicators, which is to say he provided specific body measurements, including thigh length and breast development, that, in his view, embodied normality (ibid.: Table 13). According to Pende, the normal man was strong, virile, and manly and the normal woman fertile. To be normal, a woman had to have a body shape that facilitated motherhood, for example, a large pelvis (Pende, 1939b: 381–3).^5^

Pende’s clinical experience showed that many subjects who were closest to the average type ‘very often present remarkable anomalies, especially in the genital and psychological spheres’ (Pende, 1928a: 52). A considerable part of Pende’s studies concerned these kinds of anomalies, those regarding genital development and secondary sexual characteristics. As with many scientists at that time, Pende believed that each human being was originally ‘hermaphroditic’, as embryological studies showed. The individual’s biological sex was determined by the law of heredity, and chromosomes defined the individual’s sex at the moment of fertilisation. Hormones stimulated the characteristics present in the embryo in one direction, towards either female or male. However, in an individual developing as a male, the female secondary sexual characteristics remained latent, and hormonal dysfunctions could contribute to the reappearance of these latent sexual characteristics (Pende, 1923: Vol. 1, 216–17). Anomalies in the testes’ and ovaries’ internal secretions produced what Pende called ‘genital states’ such as ‘eunuchism’, ‘eunuchoidism’, ‘virilism in women’, and ‘feminism in men’. In all these conditions, the individual acquired the secondary sexual characteristics of the opposite sex to varying degrees, and their genitals and the reproductive system could also be affected (ibid.: Vol. 2, 976–1015). Linked to these ‘genital states’ were the ‘deviations in somatic sexual development or deviations in psychosexuality’, ‘hermaphroditism’, ‘homosexuality’, ‘eroticism’, and even ‘sadism and masochism’ (ibid.: Vol. 2, 1009). According to Pende, these ‘anomalies’ were a frequent occurrence in nature and were more frequent in women than in men (Pende, 1928b: 174). Pende’s biotypology thus provided a biological classification in which hormonal deficiencies and excesses were pathologised. A taxonomy of this kind, which combined body types with specific behaviours and psychological characteristics, was designed to codify men and women as certain kinds of human being.

Pende’s taxonomy of ‘genital states’, and his view that each human being at an embryonic level was hermaphroditic, tended more to undermine the stability of the two sexes than to affirm the existence of two stable and opposite sexes. If one also considers that, as mentioned above, normality and pathology were, according to Pende, merely quantitatively different, everything contributed to give the impression that all human manifestations of biological sex fell in a continuum. Pende’s books are populated by individuals who went beyond a clear dualism of male and female, and his numerous clinical cases of ‘hermaphoditism’ or ‘intersexuality’ seemed only to confirm this point. Yet, as I will show below, hormone treatments were meant to normalise these ‘genital states’ and to restore stability. Hormones, with their capacity to modify living bodies, presented the solution and offered the opportunity to reinstate order.

But beyond Pende’s endocrine formula, or his theories and classifications of types, what is perhaps most interesting is the practical application of his work. Pende’s biotypology was not just an esoteric system of classification of types, having as it did highly practical implications and repercussions. The hormonal body, with its malleability, could be ameliorated in ways unthinkable before. Indeed, while extracts made from endocrine glands, especially testes and ovaries, had been employed for therapeutic and aphrodisiac purposes in both ancient Western and Chinese medicine, it was only in the 19th century that scientists started to experiment with these systematically on animals and human beings. In 1849, the German physician Arnold Berthold showed that testes discharged a chemical substance that affected other parts of the human body. Using roosters to carry out his investigations, Berthold revealed that after castration the roosters’ combs shrivelled, aggressive male behaviour disappeared, and they lost interest in hens. He also noticed that administering testicular extracts or transplanting testes could reverse the castration-induced changes (Watkins, 2007: 12). Some years later, in 1889, Charles Brown Séquard, a professor of medicine at the Collège de France, reported that he had experienced rejuvenation after injecting himself with extracts from the crushed testes of dogs and guinea pigs. He subsequently developed what became known as organotherapy or opotherapy, a treatment that involved transplanting animal and human endocrine glands, or injecting or orally administering extracts of endocrine glands. The transplanted or otherwise administered glands were supposed to replace the lost internal secretions naturally produced by the body (Borell, 1985: 1–20). Opotherapy reached its peak in the 1920s and 1930s, and medical doctors in the Western world came to believe that this remedy could treat virtually every kind of problem: from nervous disorders, fatigue, and menstrual pain to ageing, thyroid disorders, and diabetes. Pharmaceutical companies in the US and Europe entered the field, working with laboratories and universities to become pivotal in accelerating the process that isolated and produced synthetic hormones such as oestrogen and testosterone between the 1930s and 1940s (Oudshoorn, 1994; Sengoopta, 2006).

Historians such as Angus McLaren and Christer Nordlund have argued that hormone treatments in the interwar period came to be a way to practice eugenics (McLaren, 2007: 181–207; Nordlund, 2007). While, broadly speaking, eugenics movements have aimed to enhance the quality and/or quantity of a population, the ultimate goal being to improve its gene pool, in the interwar period eugenics took many different forms. Importantly, not all eugenicists agreed on how beneficial traits could be inherited and enhanced. Alongside the well-known Nordic eugenics, which aimed to selectively mate people with desirable hereditary traits, there existed a likewise prominent ‘Latin eugenics’, which eventually gathered formally in the Latin International Federation of Eugenics Societies in 1935 (Cassata, 2006; Stepan, 1991; Turda and Gillette, 2014). Latin eugenicists rejected the methods more typical of Nordic eugenics, such as sterilisation, and aimed to enhance the population through improvements in health, living conditions, public sanitation, education, and child welfare. Adopting a neo-Lamarckian rather than a Mendelian approach to heredity, Latin eugenicists believed that improvements in the environment could affect an individual’s health, and that these same improvements could be passed to future generations.^6^ To give a practical example, some Latin eugenicists, such as Pende, believed that changes in the individual’s environment could impact the endocrine glands, which would then secrete hormones that in turn would alter sex cells and, eventually, might be transmitted to offspring. An improved diet and more exercise, for instance, had a positive impact on hormones, and this strengthened the individual’s constitution. When factors such as diet and exercise were not enough, hormone treatments could correct the unbalanced functioning of the endocrine glands and transmit such corrections to future generations. In this sense, hormone treatments can be interpreted as eugenic forms of intervention.

Pende’s work illustrates how hormone treatments could serve eugenic purposes. Indeed, he established an entire institute that employed hormone treatments to normalise and optimise Italians. In 1925, Pende joined the University of Genoa, and in December 1926 he launched the Biotypological Orthogenetic Institute, which was a state institution (Anon., 1927: 103). Orthogenetics was a branch of constitutional medicine that dealt with issues related to individual growth; that is, with the bodily and psychological development of men and women. It relied on biotypology to ‘rationally raise and enhance’ (‘allevare razionalmente e bonificare’) human beings (Gualco and Nardi, 1941: 163). Thus, Pende’s institute had a special focus on the study and treatment of children and young adults. Aligning himself with Catholic precepts and repudiating the eugenic methods employed in the US and Germany, Pende believed that population improvements should not be undertaken through birth control or sterilisation, but only through the ‘medical normalisation of the human body and mind’ (ibid.: 159–60).^7^ Pende’s idea of orthogenesis was in line with the methods supported by Latin eugenicists. At first, the declared aim of the Biotypological Orthogenetic Institute was to screen the health and ethnic composition of the entire Italian population, and to rectify bodily anomalies and improve the Italian stock. It also functioned as a sexological centre, providing premarital counselling, evaluating racial unions, and favouring those that would, it reckoned, produce ‘fit’ offspring in the long term (Beccalossi, 2017a). Moreover, medical researchers working at the institute studied human sexual differences, assessing which constitutions were likely to prove more fertile, and how to boost the level of fecundity in women and sexual potency in men. It treated all manner of endocrinological dysfunctions, and researchers at the institute also conducted hormone tests on patients, administered various forms of therapy to stimulate hormone production, undertook experimental hormone therapies on children, men, and women, and conducted experimental research on animals (Barbara and Vidoni, 1933: 33–4). In brief, particular attention was paid to the problems of sexual development, fertility and impotence, and the study and treatment of variations in sex characteristics.

The institute consciously pursued normalisation in the sexual sphere. As Sellina Gualco and Antonio Nardi, two of Pende’s closest collaborators, wrote, ‘To improve and enhance the race we [the institute] propose the accurate control of the sexual development of the two sexes, and the biological, hygienic and, should the need arise, the medical modification of the reproductive system before marriage’ (Gualco and Nardi, 1941: 157). Men and women with ambiguous sexual characteristics, which were considered ‘constitutional inadequacies’, were corrected and normalised though a range of hormone treatments, from natural to invasive, including surgical interventions. Researchers recommended or administered a number of natural therapies such as sunlight, mountain air, and mineral water therapies, and provided guidance for special diets. These remedies were designed to stimulate the natural production of hormones in men and women. Medical researchers also offered a range of different radiation treatments, such as ultraviolet therapy, X-ray therapy, phototherapy, and Marconi therapy, which consisted of exposure to electromagnetic waves. These treatments, like the natural therapies mentioned above, were offered to stimulate hormone production, to treat certain endocrinological dysfunctions, and allegedly to improve the individual’s constitution (ibid.: 147–8). Medical researchers at the institute also offered a range of endocrinological therapies, such as opotherapy and glandular implants. Both of these treatments were employed to treat infertility in men and women, and impotence in men, and to modify the bodily characteristics of those individuals who presented ambiguous genitals and/or secondary sexual characteristics. Pende’s institute itself produced a wide range of opotherapy preparations and owned patented products manufactured by national and international pharmaceutical companies (ibid.: 146). Between the end of the 1920s and the beginning of the 1940s, the institute deployed opotherapy with extracts based on testes or ovaries extracted from pigs, bulls, calves, and other animals to treat individuals with ambiguous secondary sexual characteristics. Clinical cases of men diagnosed with ‘feminilism’, women diagnosed with ‘virilism’, and both men and women suffering from ‘eunuchism’ fill Pende’s books (see, for example, Pende, 1939a). The institute provided patients of this description with regular hormone treatments and followed their physical changes over a period of months. Therapies varied during the course of the history of the institute, depending on the technological innovations available; synthetic hormones ultimately replaced opotherapy in the 1940s. In the 1949 edition of his treatise *Endocrinology*, Pende stated that the therapy for a man suffering from ‘eunuchoidism’ consisted of the daily injection of 100–500 UR of ‘gonadostimolina’, a hormone produced by the pituitary gland that served to ‘stimulate’ the gonads. This treatment went hand in hand with the administration of 5–10mrg of synthetic testosterone every three or four days (Pende and Antognetti, 1949: Vol. 2, 577–8).

The institute also carried out surgical operations, such as mono- and heteroplastic pluriglandular implants. These implants were used to treat impotence in men, or to restore fertility in women, and were reckoned to be powerful tools for modifying secondary sexual characteristics (Beccalossi, 2017a; Pende, 1928c). These operations themselves were more than a little invasive, as they involved using fresh endocrine animal glands, removed from the animal while still alive. The surgeon transplanted the animal glands into both sides of the human testicular tissue, and, for women, into the deep tissue of their breasts (Pende, 1928c: 343). Patients generally had a fever 12 to 14 hours after the operation, which lasted for two or three days and reached 39 degrees, with other symptoms including vomiting, hypotension, and migraine (ibid.: 344). Most of the mono- and heteroplastic pluriglandular implants were followed by opotherapy extracts administered intravenously. In the first three to four months following the operation, sexual performance might deteriorate, but it would eventually improve and be normalised (Gualco and Nardi, 1941: 146). Endocrinologists such as Pende and Serge Voronoff in the late 1920s and 1930s believed that the heteroplastic pluriglandular implants could replace the lost function of the original human endocrine glands for up to three or four years (Pende, 1928c: 345). This meant that a man or a woman who had any dysfunction treated though these mono- and heteroplastic pluriglandular implants had to go through this painful procedure multiple times in the course of their lives.

As mentioned above, Pende’s output was vast, but central to his work was the study and correction of bodily anomalies. In 1929, Pende published *Anomalie della crescenza fisica. Lavori dell’Istituto Biotipologico-Ortogenetico di Genova* (Anomalies of Bodily Development: Work at the Biotypological Orthogenetic Institute in Genoa). This was a two-volume work that summarised the first two years of the institute’s activities, which focused on growth problems. The work presented about 100 case studies, most of them concerning children, teenagers, and young adults. The careful reader can detect differences in the ways in which Pende treated male and female case studies; in the case of men, Pende noted the penis volume, while he rarely commented on the size of women’s vaginas, instead noting the size of their breasts. Despite the fact that his works included tables that listed the ideal measurements for body parts (Pende: 1928a: 38-42), when presenting a clinical case Pende rarely included the exact size of the penis or breasts, limiting himself to vague descriptions such as ‘small penis’ and ‘big breasts’ (Pende, 1929[1927-1928]: 69–277).

If we compare, for example, case studies of young female and young male adults contained in *Anomalie della crescenza fisica*, these differences become strikingly evident. When writing up the cases of Emma, a 32-year-old woman from Genoa, and Giovanni, a 34-year-old man from Perugia, Pende reported their body measurements, including height, muscle mass, the chemical analysis of their blood, and their hair distribution (Pende, 1929[1927-1928]: 69-277). These were considered important characteristics in endocrinological works of the time, as they were used to corroborate whether the secondary sexual characteristics of the individual were typically male or female. Pende observed that Giovanni’s pubic hair had a ‘feminine distribution’, his testes were ‘flaccid’, and the penis developed. Yet when it came to Emma, he offered no description of her genitalia, only her breasts, which were described as ‘big’ (‘abbondanti’). He also recorded whether Emma and Giovanni masturbated and when they had first had sexual intercourse. In both cases, Pende’s treatment aimed to correct the secondary sexual characteristics, but only in Giovanni’s case was the opotherapy’s effect on his genitals deemed relevant.

## Argentina, biotypology, and the normalisation of the body

Biotypology came to inform much of the thinking about sexual and racial differences in Latin America and Southern Europe in the interwar period, with Argentina perhaps the Latin American country with the closest links to Italian biotypology. As Yolanda Eraso has pointed out, Argentinian enthusiasm for Italian biotypology in the 1930s stemmed from a common tradition of scientific thought that adhered to Lamarckian principles. Medical professionals in the two countries likewise shared the belief that it was possible to provide environmental interventions to improve their respective populations’ health, which in turn resulted in medical doctors’ advocacy of government intervention in areas such as hygiene and social medicine. Italian migration to Argentina also served to maintain strong cultural ties between the two countries. In 1914, the Argentinian census found that half the population in Buenos Aires was of foreign extraction (mainly Italians and Spaniards; Eraso, 2007: 796). Since the end of the 19th century, a number of Italian medical doctors had migrated to Argentina for political reasons, and it was common for Argentinian doctors to spend some time in Europe, quite often in Italy, to advance their careers. In addition, the Italian fascist regime cultivated close ties with Argentina and supported institutes and various activities with the aim of spreading Italian culture in a country that was sometimes seen as an ‘informal colony’ (Finchelstein, 2010: 35; Reggiani, 2010).

As in Italy, so too in Argentina hormone treatments came to be seen as an effective tool of eugenic intervention (Beccalossi, 2017b). Taking inspiration directly from Pende’s institute in Genoa, the physician Arturo Rossi founded the Instituto de Biotipología, Eugenesia y Medicina Social (Institute of Biotypology, Eugenics, and Social Medicine) in Buenos Aires in 1931 (Rossi, 1944: Vol. 1, 39). Rossi had taught at and been the director of the Medical Clinic in the Faculty of Medical Sciences at the University of Buenos Aires before devoting his career to biotypology. Following a Ministry of Foreign Affairs and Worship decree, Rossi spent 13 months in Europe studying biotypology, mainly in Genoa with Pende (Vallejo and Miranda, 2011: 63).^8^ Upon his return to Buenos Aires in February 1932, Rossi, with Donato Boccia and Octavio López, founded the Asociación Argentina de Biotipología, Eugenesia y Medicina Social (Argentinian Association of Biotypology, Eugenics, and Social Medicine; Rossi, 1944: Vol. 1, 16). One of the association’s main purposes was to study constitutional medicine and the application of its principles in order to identify the Argentinian ethnic biotype and to develop an appropriate eugenic programme (Palma and Gómez Di Vincenzo, 2009: 6). The following year, in 1933, the *Anales de Biotipología, Eugenesia y Medicina Social* (Annales of Biotypology, Eugenics, and Social Medicine) was founded, and this important journal was headed in turn by Rossi, López, and Gonzalo Bosch. The *Anales* was published every two weeks and functioned as an important hub for the circulation of biotypological ideas between the Northern and the Southern hemispheres. Pende, and other Southern European endocrinologists such as the Spaniard Gregorio Marañón, regularly contributed articles to this journal. In 1934, the association founded the Escuela Politécnica de Biotipología, Eugenesia y Medicina Social (Polytechnic School of Biotypology and Allied Matters), where students were trained in ‘biological eugenics and puericulture’ and in ‘legal and social eugenics’. Finally, in 1943, the Argentinian minister of justice and public education, Dr Guillermo Rothe, officially created a chair of biotypology in Buenos Aires and started a process that led to the nationalisation of the Instituto de Biotipología, Eugenesia y Medicina Social, which ultimately occurred in 1944 under a later minister of justice and public education, the medical doctor Gustavo Martínez Zuviría. Rossi led this new phase of the institute, which was renamed the Instituto Nacional de Biotipología y Materias Afines (National Institute of Biotypology and Related Subjects), and the School of Biotipology in Buonos Aires continued to train hundreds of graduate students each year until at least the 1960s (Rossi, 1944: Vol. 1, 17).

Rossi, the most ardent advocate of biotypology, published a number of articles on its basic principles in the first issues of the *Anales*, specifically addressing the concept of normality in the 15 April and 15 May 1933 issues (Rossi, 1933a, 1933b). In the 15 May 1933 issue, Rossi stated that the Argentinian School of biotypology followed the Italian School in its method, with a view to establishing the average type (‘tipo medio’), highlighting in particular the importance of Pende’s concept of ‘racial normality’ and ‘regional normality’. As mentioned above, Pende believed that the normal type could be identified only within an ethnically homogeneous region. While Rossi agreed with Pende’s observations about the non-existence of a pure race, he did point out that Argentina was much more ethnically diverse than Italy. It was founded on Hispanic and Italian migrants, but there were immigrants from other parts of the world, along with indigenous populations. This ethnic mix presented a challenge when biotypologists set out to search for the normal Argentinian biotype. Rossi suggested that when selecting individuals to study, one needed to select those who had descendants from the same ethnic group. Only families that had not mixed with other races for a number of generations could be considered valid subjects when analysing the ‘normal type’ (Rossi, 1933b: 13). In his *Tratato*, published in 1944, Rossi, adopting a vocabulary that was very similar to Pende’s, suggested that biotypology and orthogenesis could lead to the enhancement of the individual and of the race (‘bonificación humana del individuo y de la raza’; Rossi, 1944: Vol. 1, 159). He explained what he meant by race: it was not only defined by the common physical characteristics of a large proportion of the population, but also included the social and ‘spiritual’ heritage, in other words, the culture people shared (ibid.: Vol. 1, 240). In line with the racism typical of Southern Europe, Rossi argued that while miscegenation was a positive occurrence, the mixing of ‘very different races’, such as white and black, was detrimental for a country (ibid.: Vol. 1, 122–3). In particular, he argued that the Argentinian race had improved with the disappearance of the indigenous and black populations (ibid.: Vol. 1, 149).

If the normal type was an ideal average and if only a tiny minority of individuals would fit within these standard measurements, normality could be pursued for one and all through the application of the principles and methods of biotypology and orthogenesis. In Rossi’s view, orthogenesis was a form of eugenics, but differed insofar as it was a post-natal intervention. ‘Orthogenesis’, he explained, was ‘the normalisation of [the] human factory [meaning the human body]…through any means in order to obtain harmonious normality, [the] foundation of a strong constitution from both a bodily and [a] psychological perspective’ (Rossi, 1944: Vol. 1, 156). Orthogenesis focused on children and young adults in particular, and Rossi also defined it as the ‘science of the child’s constitution, with the aim to normalise it morphologically, morally and intellectually’ (ibid.: Vol. 1, 12). He then highlighted the political and medical importance of biotypology, explaining that it was taught in the most important universities in the world, and that it could be applied in the widely varying fields of ‘race improvement’ (‘bonifica della razza’), social medicine, hygiene, and clinics (ibid.: Vol. 1, 39).

While Rossi believed that a number of recent government policies, such as matrimonial certificates, had proved to be useful, hormone therapies were the practical and effective eugenics tools to ‘correct’ all individuals who deviated from the norm. This meant that all individuals who presented secondary sexual characteristics typical of the opposite sex, as in the case of ‘eunucoidismo’, and who did not present the characteristics of a ‘normosexualidad’ (normal sexuality), could be treated (Bonorino Adaondo and Rossi, 1933: 5). Although Rossi had a remarkable understanding of endocrinology, and wrote a great deal about recent hormone research, and although his institute included an endocrinological dispensary from 1939 (Anon., 1939), Rossi himself appears not to have conducted any experiments with hormone treatments.

Significantly, in the 1930s, just as biotypology was flourishing in Argentina, physiological institutes, headed by the first Argentinian Nobel Prize winner, Bernardo Houssay, grew rapidly and achieved an international reputation. Crucial to this expansion was the significant economic support from the Rockefeller Foundation, which was interested in financing studies on endocrine hormones ‘that could illuminate issues of growth, body activities, behavior, old age and sexuality’ (Eraso, 2007: 801). Within this context, Mariano R. Castex, professor of clinical medicine at the University of Buenos Aires and president of the National Academy of Medicine, was active in biotypological circles and carried out hormone therapies to normalise bodies. In 1930, Castex invited Pende to give a series of lectures in Buenos Aires and between 1932 and 1942, he was the president of the Argentinian Association of Biotypology (Rossi, 1944: Vol. 1, 16). He published on a number of topics, from syphilis to digestive problems, and employed biotypological taxonomies to classify the individuals he treated. Between 1920 and 1940, he also published a series of clinical cases of people who presented ambiguous secondary sexual characteristics, individuals who were classified as ‘intersexual’, women who were going through menopause, and men suffering from impotence, all of whom were treated with hormone therapies. Castex’s work clearly offers an example of how biotypologists in Argentina normalised people whose bodies did not conform to the standard of what being a man or a woman meant.

Among his published cases are studies of what he called ‘feminilism’, that is, the condition of individuals who had a penis and secondary sexual characteristics that were typical of women. For example, in 1927, he published three clinical cases in *La Prensa Medica Argentina*. The first case, a study of a ‘diecenphalic feminilism’, was the most detailed, and describes the circumstances of José M., a 27-year-old single lithographer with an extremely weak constitution (Castex and Camauër, 1927a: 759–60). When he was 16 years old, José started to grow extremely tall, due, Castex hypothesised, to functional disturbances in the ‘diecenphalic’ growth centre, the pituitary gland, or the testicular interstitial gland (ibid.: 760). By the time he was 18, José was 1.97m tall and starting to masturbate and have sexual intercourse with women on a weekly basis. Commenting on José’s sexual life, Castex specifically stated that ‘for some men who are 18 years old, sexual intercourse once a week is normal, for most of them it is not enough’. By the time José was 22, however, his sexual apparatus had stopped working properly after a traumatic accident in which he had suffered a serious fall. When Castex examined José at 27 years old, he highlighted that José’s pubic hair had ‘the classical feminine pattern’. Following his examination, Castex observed that José did not have much body hair for a man who was 27 years old, that he showed the ‘[secondary] sexual characteristics of the opposite sex’, and that he could be identified as a case of ‘hipogenital eunuchoid of the feminine type’ (‘hipogenitalismo eunocoide a tipo feminino’; ibid.: 761–2). Castex’s diagnosis was that José suffered from ‘diabetes insipidus’, ‘disgenitalism’, ‘feminilism’, and ‘psiquism’, and he further stated that he had successfully treated the diabetes with opotherapy from pituitary gland extracts (ibid.: 768–9).

Specifying the kind of eunuchoidism José manifested, Castex argued that he was a case of ‘pure eunuchoidism with feminilism’: although his genitals were originally well shaped, they had atrophied as in the case of ‘late eunuchoidism’. His pelvis was feminine, as was his face (Castex and Camauër, 1927a: 769). Castex explained that originally the embryo was ‘bisexual’, meaning that it was sexually undifferentiated and that it had the potential to develop in either direction, although one sex would develop while the other remained latent. Most conditions when an individual displayed secondary sexual characteristics typical of the other sex could be explained through this latent presence, which somehow re-emerged. According to Castex, José’s body indicated the ‘first degree of sexual inversion and of constitutional feminilism’ (‘feminilismo constitucional’; ibid.: 761). In this clinical case, Castex was not clear on whether ‘sexual inversion’ referred to homosexual behaviour or exclusively to José’s feminine secondary sexual characteristics.^9^ In the 1930s, a number of endocrinologists and medical scientists continued to use the term *sexual inversion* to indicate either same-sex desires or the presence of secondary sexual characteristics typical of the opposite sex. In his influential *La Evolución de la Sexualidad y los Estados Intersexuales* (The Evolution of Sex and Intersexual Conditions, 1930), Marañón interpreted a number of conditions, from ‘hermaphroditism’ to ‘sexual inversion’, as part of the same phenomenon. Although ‘hermaphoditism’ in Marañón’s work referred to what we now call intersex variations, and ‘sexual inversion’ to homosexuality, both were classified as ‘intersexual conditions’ whose origin was explicable through the working of hormones (Cleminson and Vázquez García, 2009: 155–6; Glick, 2005). A number of Latin American scientists tended to interpret hormone dysfunctions as the cause of intersex variations and homosexuality, and sometimes they even conflated the two ‘conditions’. Interestingly, despite the fact that Castex reported how often José masturbated or how many times a week in the past he had had sexual intercourse, he was not interested in expanding his observations about the sexual instinct. Indeed, it is likely that in this article Castex used the term ‘sexual inversion’ to indicate the presence of secondary sexual characteristics in José that were typical of women. Castex seemed to use hormone treatments simply to make men more virile, and he did not make any reference to José’s homoerotic desires. He must have been aware that effeminacy in men was often associated with homosexuality, and biotypologists in Argentina sought to eradicate homosexuality, but we cannot infer that his patient was homosexual (Castex and Camauër, 1927a: 761). Castex focused on the body and had little interest in the sexual instinct. In Argentina, as was the case in Italy, hormone treatments were used to cure people whose bodies did not conform to medical standards of normality.

José’s eunuchoidism was treated with pluriglandular opotherapy of thyroid, surrenal glands, and testes, through both injections and glandular grafts (Castex and Camauër, 1927a: 773). According to Castex, injections of testicular extract made men more masculine, while injections of ovary extracts rendered women more feminine (Castex and Camauër, 1927b: 794). While Castex did not enter into too many details about the modalities of hormone treatments in the articles he published in 1927 in the *Prensa Argentina*, in other publications he was more forthcoming. For example, in 1920, Castex published another case of ‘feminilismo eucunoide’, where he had injected extracts made from animal testicles, thyroid, and surreal glands to render the subject more virile (Castex and Waldrorp, 1920). He used the ‘extractum testis’, testicle extracts from the ‘Zimasa company’, an Argentinian pharmaceutical company that had laboratories in Buenos Aires (ibid.: 295). The Argentinian *Anales* regularly advertised Zimasa’s hormone treatments during the 1930s, and these advertisements indicated that Zimasa products were used for a range of different conditions: dysfunctions of the testes, eunuchoidism and castration, underdeveloped testes during puberty, impotence, and a lack of sexual vigour when ageing. For example, Zimasa sold 30g ampoules made of cock testes to be injected on a daily basis, and scrutiny of *Anales* volumes would suggest that these kinds of hormone product were still being sold in Argentina up until the end of the 1930s (Instituto Endocrino Zimasa, 1939: 14). In Argentina, as in Italy, biotypologists such as Castex believed that treatments such as opotherapy or the grafting of animal endocrine glands were an effective way to therapeutically manipulate the body. The aim was to restore order and bring individuals towards the standard of the male or female sex.

## Brazil, biotypology, and the normalisation of the sexual instinct

While biotypology in Italy started to decline with the end of the Second World War, when the Italian government removed Pende from his teaching post due to his collaboration with fascism, it continued to grow and receive institutional support in Latin America. In the 1930s, the biotypological orthogenetic file or ‘biotypological card’, a document developed by Pende to control the Italian population, was adopted in Argentina, Brazil, and Mexico.^10^ In the 1940s, biotypology was taught in a number of universities throughout Latin America and continued to be taught after the Second World War. In Brazil, biotypology did not receive as much institutional support as in Argentina, but it was nevertheless adopted in medical circles, and was taught in medical schools, with a number of publications devoted to the subject.

As historians Ana Carolina Vimieiro Gomes and André Luiz dos Santos Silva have pointed out, Brazilian biotypologists ‘creatively selected’ the most important theories and techniques for corporeal classification coming from both Europe and the US. Among these appropriations, the Italian approach was dominant in Brazil; Italian biotypologists provided the main framework and were particularly esteemed for their supposedly scientific precision in classifying ‘normal’ and ‘abnormal’ bodies (Vimieiro Gomes and dos Santos Silva, 2019: 84). Significantly, Italian biotypological classifications, with their ‘normalising lexicon, were seen as an alternative to traditional racial typologies’ (ibid.: 82). Yet such an adoption of Italian theories should not be seen simply in terms of Southern European influence upon Brazil. As a number of historians have noted, Latin American scientists in the interwar period had an eclectic approach to European science, especially when it came to reinterpreting racial and sexual categories (Jones, 2017).

Rio de Janeiro in particular became a centre for biotypological studies. The University of Rio de Janeiro had an important school of constitutional medicine and biotypology led by Juvenil Rocha Vaz and Waldemar Berardinelli, both professors at the Faculty of Medicine (Rossi, 1944: Vol. 1, 38; Vimieiro Gomes and dos Santos Silva, 2019: 82). In the early 1930s, the University of Rio de Janeiro also established a Laboratory of Biotypology that was linked to the teaching of compulsory clinical courses for first-year students. In this laboratory, biotypologists conducted research with the aim of determining the supposed ‘Brazilian normotype’ and explored the relationship between an individual’s biotypological profile and the development of diseases (Vimieiro Gomes, 2017: 147). In 1932, writing in the *Archivos do Instituto Medico-Legal* (Archives of the Legal-Medical Institute), Berardinelli wrote an article entitled ‘The Concept of Normality: The Personality or the Biotype’. He noted that the concept of normality was ‘highly relative’ in biology. On the one hand, normality was an ‘abstract idea’, as De Giovanni’s work on the ideal constitution had demonstrated. On the other hand, it was a concept based on a numerical standard, as in Viola’s studies on constitutions, which had applied Quetelet’s statistical method, whereby the average type resulted from the central values on the variation distribution curve (Berardinelli, 1932: 80).^11^ As Berardinelli clarified, Viola’s concept of the ‘average-normal type’ (‘tipo médio-normal’) had had some value, but in nature true individuals excluded normality and there were only infinite variations of individuals (ibid.: 81).

If Berardinelli highlighted that human diversity in general did not conform to the ideal standards of science, other Brazilian biotypologists were for their part concerned with identifying the shortcomings of Italian classifications, which could not be expected to reflect Brazilian racial diversity. Indeed, while the Italian biotypological classifications appeared more flexible than traditional racial classifications adopted in Northern Europe and North America, Brazilian biotypologists still faced the problem of how to apply European biotypology in Brazil, where the extent of racial variety made it virtually impossible to find an average type. In 1934, a doctor by the name of Isaac Brown published *O Normotypo Brasileiro* (The Brazilian Normal Type), a re-elaboration of his PhD thesis undertaken while working at the Gabinete de Biotipologia at the Faculty of Medicine of Rio de Janeiro, under the supervision of Juvenil Rocha Vaz. The book was dedicated to Brown’s *maestri*, Rocha Vaz and Berardinelli, and was awarded the Prêmio de Medicina pela Sociedade de Medicina e Cirurgia do Rio de Janeiro. Using Viola’s anthropometric method and Quetelet’s statistical methods, Brown aimed to find the Brazilian normal type (Brown, 1934: 13, 24).

Brown studied 702 healthy Brazilians between the ages of 20 and 50, with Brazilian-born descendants going back at least four generations. He admitted that a statistical method could be applied only when large data sets existed and that he had found it difficult to collect enough individuals to properly apply a statistical analysis. He then explained that the subjects he had studied could be divided into two groups: the first were his friends or acquaintances, whose occupations ranged from lawyers and traders to day labourers; a number of firemen constituted the second group. Brown had specifically chosen to study firemen as they had to be fit and healthy to be able to do their job (Brown, 1934: 86–90). Another problem Brown encountered was how to divide up the ethnic composition of his chosen subjects. Following Dr Roquette Pinto’s study, he divided his subjects into white or ‘leucodermos’; mulattos or ‘phaiodermos’ (individuals resulting from mixed couples, white and black); ‘caboclos’ or ‘xanthodermos’ (individuals resulting from mixed couples, white and Indian); and black or ‘melanodermos’. He also divided Brazil into four regions, and analysed white people separately, as he felt he had enough white subjects; only 108 subjects studied were black (ibid.: 101, 104–5). Brown admitted that if one were to follow Viola’s method in order to find a normal type, one would be forced to conclude that no one in Brazil was normal. The racial composition was too varied, and he could only identify biotypological variations that, when aggregated, made up the overall combination of the Brazilian population (ibid.: 143–51; Vimieiro Gomes, 2017: 149–50). The single Brazilian ‘normotype did not exist in nature’, Brown concluded (Brown, 1934: 142–53).

If the normal type did not exist, biotypology was still used to normalise individuals in Brazil. Indeed, Brazilian sexologists welcomed biotypological thinking and used hormone therapies to modify ‘deviant’ sexual behaviour. By the late 1920s and early 1930s, sexology was a well-established field in Brazil, and a significant number of books on sexual matters had been published and were available to a lay audience (Ford: 1995: 48). In Brazil, biotypology was incorporated into criminal anthropology, a discipline that had traditionally been concerned with so-called ‘sexual perversions’. For example, Leonidio Ribeiro, a Brazilian criminal anthropologist who was also well known outside Latin America for his studies on male prostitution, published a work in 1938 in which he employed Pende’s biotypological thinking to study male homosexuality and advocated the use of hormone therapies to treat homosexuals (Ribeiro, 1938). But Ribeiro was not an endocrinologist, and while he came to believe that hormone treatments represented a solution, he simply reported the endocrinologists’ therapies as promising.

The celebrated sexologist Hernani de Irajá provides evidence of how sexologists employed hormone therapies to normalise people and their sexual desires. Irajá obtained a degree in medicine in Porto Alegre (Rio Grande do Sul) before moving to Rio de Janeiro, where he became well known for his sexological writings, many of which targeted a popular audience. His book *Psychoses do Amor* [Psychoses of Love], first published in 1917, went through 17 editions, and he worked as a private sexologist, receiving patients who came to see him in the hope of resolving their sexual ‘problems’, from impotence to homosexuality (Russo *et al.*, 2011: 35–6). He was the director of the first specialised journal on andrology, the *Jornal de Andrologia* (Journal of Andrology, 1932–8), and of the *Boletim de Educação Sexual* (Bulletin of Sexual Education, 1933–9), a journal that targeted a middle-class public (Carrara and Russo, 2002). In his early works, Irajá did not use biotypology, although he increasingly relied on it during the 1930s. For example, in 1937, he published *Tratamento dos males sexuales* (Treatment of Sexual Disorders), which was clearly influenced by biotypology, as the labels adopted to describe his patients indicate. When expounding his clinical cases, he employed categories such as brevilineal and longilineal constitutions. Irajá took normal sexuality to be reproductive sexuality, and in this book he openly spoke about hormone treatments that he had administered in an attempt to treat ‘sexual perversions’, including homosexuality. According to Irajá, anomalies of the sexual instinct could be hereditary (biological), acquired, or a combination of hereditary and acquired. In his *Tratamento dos males sexuales*, he reported that in his career he had so far observed 100 cases of homosexuality, 70 male and 30 female, and that he had attempted to treat them through hormone therapies (Irajá, 1937: 223). To give some examples of what such therapies entailed, he published five case studies in which he described the range of treatments offered. The first case, A. F., was a 21-year-old man whose sexual inversion was accompanied by ‘gynaecomasty’, an endocrine condition in which a man’s chest assumes the volume typical of a woman’s breasts. The subject had a longilineal and ‘female constitution’ and had started to masturbate when he was eight years old, and when he started boarding school at 12 he had begun practicing ‘sodomy’ with his classmates, as the passive partner (ibid.: 210–11). Irajá treated him with galvanic electrical stimulation, putting the negative pole on the patient’s penis and the positive pole on the anus. He also employed opotherapy – Irajá carried out 120 injections of testes and prostrate extracts – and employed gymnastics, massage around the thyroid area, and psychoanalysis. Although these treatments lasted four months, Irajá admitted that he had failed to ‘cure’ A. F. (ibid.).

The second case of male sexual inversion did not display feminine secondary sexual characteristics. The 29-year-old patient was originally from Germany and had an ‘athletic normo-type’ constitution, with normal body hair and a masculine voice and genitals. However, since childhood he had enjoyed wearing his sisters’ clothes, which sexually aroused him. Irajá’s treatment lasted three months, and the patient received a combination of 51 hormone treatments, psychoanalysis, and hypnosis. This case proved more successful than the first, as the German patient started to have sexual intercourse with women, although he was still attracted to men (Irajá, 1937: 213–14).

The third male sexual invert was a 68-year-old ex-soldier who was ‘almost normal’. His constitution was fine, but from time to time, generally when he had haemorrhoids, he could not resist having sexual intercourse with other men. His treatment consisted of a broad range of therapies, including bathing his genitals at 45 degrees twice a day, exposure to ultraviolet light, and galvanic electrical stimulation of the erogenous zones. For three months, this patient also received testes, prostrata, and renal cortex extracts, although all of these treatments proved unsuccessful (Irajá, 1937: 215–16).

Irajá also reported two cases of female sexual inversion. The first was L. S., a 25-year-old woman, single, with a ‘brevilineal’ constitution but masculine attitudes such as an aptitude for business and maths. She had not developed the secondary sexual characteristics typical for her age. She started to feel attracted to women when she was 10 years old, although at that age her attraction to members of the same sex was platonic. She began to menstruate at 17, and at that age she began having sexual intercourse with other women, as both an active and a passive partner. Irajá treated her with 40 injections of ‘gynhormon 50 u.r.’ ampoules (‘ampolle’) and 20 ampoules of hyphophists. While this led her to put on weight, her attraction to women continued. Irajá subsequently began a daily diathermic application to her hips to affect her ovaries, and while Irajá noticed some physical improvements, her homosexual inclinations did not change (Irajá, 1937: 217–18).

The second female case was a 40-year-old Italian who had a brevilineal constitution. While she possessed some facial hair, she was in fact married to a man, although she had felt attracted to women since she was 18 years old and had also had sexual intercourse with a range of different women. Irajá first treated the patient with 15 and then 30 thyroid extracts, along with a regime of general rest (Irajá, 1937: 219). Her bodily condition improved, and Irajá then started to administer her testes extracts, although again the patients’ homosexual inclination did not disappear (ibid.: 222). Irajá was sceptical about the results of hormone therapies because while he could observe bodily improvements, homosexual desires did not disappear. Out of more than 80 cases, only in 12 did Irajá notice improvements (ibid.: 216–17). Yet, despite such a low success rate, he clearly used hormone treatments in his attempts to modify his patients’ sexual behaviour.

## Conclusions

As the historian of science Jean-Paul Gaudillière notes, ‘Normalizing human bodies is…always linked to the ability to define boundaries between the normal and the pathological’ (Gaudillière, 2004: 528). Yet in the medical thinking that stemmed from constitutional medicine and continued with biotypology, the definition of normality remained vague and abstract. In part, the inability to define normality came from the intellectual shortcomings of the 19th-century statistical methods that were at the basis of constitutional medicine. The ‘normal’ biotype described by Pende and other biotypologists on both sides of the ‘Latin Atlantic’, with *all* primary and secondary sexual characteristics falling into the parameters of a standardised normality, did not exist in nature. When biotypologists set out to study the normal type in Latin America, where the racial composition was less homogeneous than in Southern Europe, 19th-century statistical methods and biotypological reasoning proved inadequate. Yet, with all the failings of their methodology, advocates of biotypology were so preoccupied with the numerical and the aesthetic parameters of the normal type that they ended up deriving endless taxonomies of variations from the norm in their attempts to define the normal type.

While biotypology provided a tentative scientific foundation for arguing that a person did not match the ideal or normal type, endocrinological research made available the practical tools for normalising individuals who did not match the standard. Displacing the physiological model that had held sway in 19th-century medical thinking, in the 20th century hormone research promoted an understanding of the body in which ‘hermaphroditism’, homosexuality, and other ‘sexual perversions’ were all attributed to anomalies in the internal secretions produced by the testes or the ovaries. While the Argentinian Castex aimed to alter the physical characteristics of men with effeminate bodies, Brazilian sexologists such as Irajá employed hormone therapies explicitly to alter the sexual instinct. Medical techniques of normalisation such as hormone therapies, offering as they did post-natal interventions, conformed with Catholic precepts. Yet, as the Italian Orthogenetic Biotypological Institute made evident, such therapies were invasive eugenic treatments devised to improve the population.

Finally, in the 20th century a number of new medical technologies were created, hormone treatments among them. Hormone treatments as carried out in the interwar period in Southern Europe and Latin America were designed to re-establish normality. When biotypologists and sexologists treated intersex and gay people with hormones, they aimed to restore standard male and female sexual characteristics, and heterosexual reproductive sexual behaviour along with them. Hormone treatments, in the practices analysed in this article, were designed to bring an individual closer to normality, whatever that was supposed to mean. Since the Second World War, the same medical technologies have increasingly made it possible to alter established and unquestioned concepts of normality. While there is still a trend towards using hormone treatment to normalise individuals and to bring their bodies to a state that is aligned with the accepted norms of male and female bodies, increasingly hormone treatments have offered possibilities of enhancement, experimentation, and even contestation. Hormone treatments, as embraced by some trans people, are defying binary views of sex, gender, and norms. When a trans philosopher such as Paul Preciado declares in their book *Testo Junkie* that they initially started to take testosterone not to transition, but because they wanted to experiment with their body, this is indicative of how the same medical technologies that were intended to normalise people are now opening up a way to contest norm and normalisation alike (Preciado, 2013). It is perhaps a paradox that it was specifically the interwar constitutional doctors, biotypologists, and endocrinologists who were highly aware that normality did not exist in reality (or was an extremely rare phenomenon) who so vehemently wanted to normalise individuals.

## Notes

I would like to thank Peter Cryle and the anonymous peer reviewers for their comments on a previous version of this article. Any remaining mistakes are my own.

1. My larger project focuses on the mutual exchange between Italy, Argentina, and Brazil. This is not the place to explore how and why medical knowledge travelled back and forth between these three specific countries, but Argentinian biotypologists such as Arturo Rossi and Brazilian biotypologists such as Walter Berardinelli, to name just two, were discussed by Italian biotypologists. I have found less evidence that Mexican biotypologists had an impact on Italian science.

2. For a historical account of the actual techniques of normalisation though hormones and the institutions that carried out hormone treatments, see Beccalossi (2020).

3. The italics in this quotation are in block capitals in the original text. All translations are mine unless otherwise stated.

4. On theories of race in Italian science and eugenics in the interwar period, see Gillette (2002).

5. In *Trattato di biotipologia umana*, Pende identified four types of normal woman, all of whom had a body shape that facilitated motherhood.

6. As is well known, Lamarck believed that acquired characteristics could be passed on to offspring.

7. The original Italian reads as follows: ‘la normalizazzione medica della fabbrica umana fisica e psichica’.

8. The Ministry of Foreign Affairs and Worship (Ministerio de Relaciones Exteriores y Culto) was the ministry that oversees the foreign relations of Argentina.

9. The authors described José as a ‘degenerate’, a term that could indicate homosexuality.

10. On the influence of Italian biotypology in Latin America, see Stepan (1991).

11. On Quetelet, see Cryle and Stephens (2017: 100–28).
